# Changes in Shrimping Effort in the Gulf of Mexico and the Impacts to Red Snapper

**DOI:** 10.1016/j.isci.2020.101111

**Published:** 2020-04-27

**Authors:** Benny J. Gallaway, Scott W. Raborn, Laura Picariello, Nathan F. Putman

**Affiliations:** 1 LGL Ecological Research Associates Inc., Bryan, TX 77802, USA; 2 Texas Sea Grant, Texas A&M University, Corpus Christi, TX 78412, USA

**Keywords:** Environmental Science, Ecology, Biological Sciences

## Abstract

Despite a complex management landscape and decades of overfishing, Red Snapper (*Lutjanus campechanus*) stocks have grown substantially in the Gulf of Mexico and restrictions on the fisheries that catch them are being loosened. This year, annual shrimping effort was allowed to increase by 21% after National Marine Fisheries Service scientists concluded that the resulting bycatch of Red Snapper would only reduce the annual allowable catch in other fisheries by ∼1% and have no impact on population growth. Nonetheless, the recreational fishing sector intensely campaigned against this rule, fueled by wild mischaracterization of shrimp trawl bycatch in media outlets targeting anglers. Here, we aim to elevate the debates surrounding Red Snapper management by presenting scientific and historical context for the potential impacts from shrimping. We discuss our views of the current problems facing Red Snapper and key ecological questions to address for more effective management of this resource.

## Graphical Abstract

**Figure:**
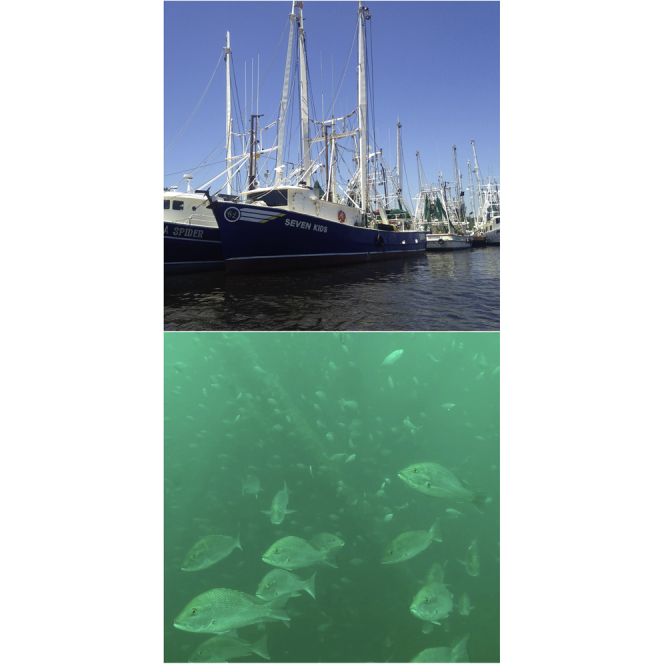

## Background

**Figure 1:**
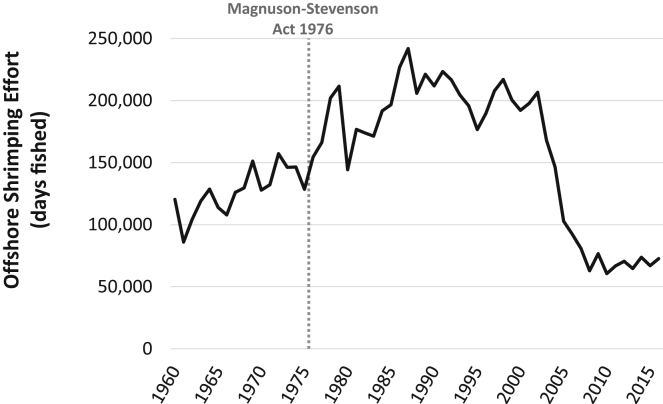
Annual Fishing Effort in the Offshore US Gulf of Mexico (1960–2016)

Gulf of Mexico Red Snapper (*Lutjanus campechanus*) is one of the most important and controversial species fished in the United States and is at the nexus of three multimillion-dollar industries: directed recreational fisheries, directed commercial fisheries, and bycatch in commercial penaeid shrimp trawls (Gallaway et al., 2017). By the late 1980s there were indications that the population was overfished, and the belief was that the primary problem was lack of juvenile recruitment due to bycatch in shrimp trawls (Goodyear, 1994). Thus, nearly all management effort to rebuild the stock was directed at reducing bycatch through the modification of shrimp trawls (bycatch reduction devices, BRDs). Directed harvest in commercial and recreational fisheries were managed by reducing the length of the fishing season, size limits, licenses (commercial and for-hire), and bag limits (recreational). Despite these actions, the annual quota for these fisheries tripled between 1990 (3.1 million pounds) and 1996 (9.12 million pounds), where it remained through 2006 (de Mutsert et al., 2008). Between 1990 and 2002, Gulf-wide offshore shrimping effort was relatively stable, averaging 203,085 (range: 176,589–223,388) fishing days (a “fishing day” equals 24 h of actual shrimp trawling by one vessel). However, by 2003, independent of Red Snapper management, shrimping effort dropped to its lowest levels since 1980 (Figure 1). Shrimping effort continued to fall thereafter, dropping to 92,372 days fished by 2006. Over this period the Red Snapper resource showed limited population growth.

With hindsight, the reason for the lack of recovery was several-fold. BRDs were less effective at precluding Red Snapper than initially estimated (Gallaway and Cole, 1999), but perhaps most important was that the natural survival rates of young Red Snapper were much lower than previously believed, and thus the influence of bycatch was relatively small (Gazey et al., 2008, Gallaway et al., 2009). Essentially, if the fish had not been caught in shrimp trawls most would still have likely died before reaching reproductive maturity and entering the directed fisheries. Present estimates of natural mortality indicate that only ∼5% of Red Snapper bycatch would have survived to 2 years old. In contrast, annual survival rates of Red Snapper ≥2 years of age exceed 80% (Putman and Gallaway, 2020). Thus, protecting a few older Red Snapper (caught in the directed fisheries) is more beneficial to the population than protecting many younger fish (caught as shrimp trawl bycatch).

## A Shift in Management

**Figure 2:**
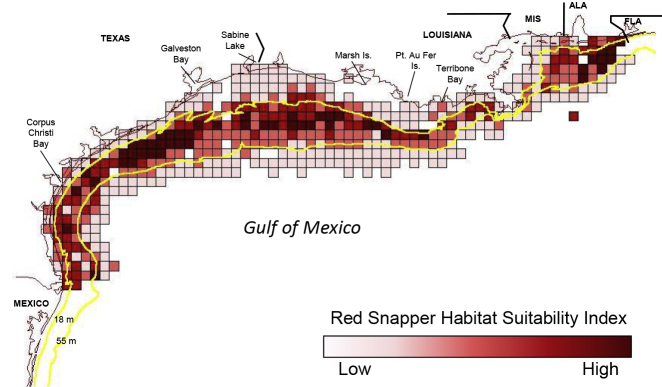
Red Snapper Index Area in the Western Gulf of Mexico From Texas to Alabama, the area between 10- and 30- fathom (approximately 18 and 55 m) depth contours is designated as an index area for Red Snapper (between the yellow lines). This area encompasses high-value habitat for juvenile Red Snapper (Gallaway et al., 1999). Habitat Suitability Index (HSI) values are indicated by shading; darker shades represent better Red Snapper habitat. Shrimping effort in the index area is managed to limit juvenile Red Snapper mortality from shrimp trawl bycatch.

With that new ecological information on Red Snapper survival rates, management efforts began to include limiting the catch of commercial and recreational directed fisheries that targeted the mature and highly fecund fish (Gallawayet al., 2017). Amendment 27 to the reef fish FMP decreased the total allowable catch of Red Snapper in the directed commercial and recreational fisheries from 9.1 million pounds to 6.5 million pounds in 2007 and then 5.0 million pounds in 2008 and 2009—a 45% reduction (GMFMC (Gulf of Mexico Fishery Management Council), 2007). Also in 2007, Amendment 14 established an index area to protect juvenile Red Snapper from unsustainable levels of bycatch mortality in shrimp trawls (GMFMC (Gulf of Mexico Fishery Management Council), 2007). The index area is located in the 10- to 30-fathom depth (18–55 m) region of the Gulf of Mexico, extending from Alabama west to Mexico (Figure 2).

In this index area, the amount of shrimp fishing effort has shown to be a good measure of Red Snapper mortality from shrimp trawl bycatch (Gallaway et al., 1999). Shrimp fishing effort in the index zone was reduced by 74% of the average total effort from 2001 to 2003 (82,811 days fished) (GallawayGazey and Cole, 2017). Thus, by 2008, shrimp fishing effort in this zone could not exceed 21,531 days. If this level was reached or exceeded in a given year, a seasonal closure of the shrimp fishery would be triggered. For context, Gulf-wide shrimp effort from 2007 to 2016 has averaged 69,554 days fished (a 66% reduction from the 1990–2002 period) (Figure 1).

As the Red Snapper population grew, the 2007 amendments (GMFMC (Gulf of Mexico Fishery Management Council), 2007) were adapted to allow increases in the number of Red Snapper removed by all sectors—as long as the rebuilding 2032 deadline was not jeopardized (GMFMC (Gulf of Mexico Fishery Management Council), 2007). This included both increased catch in the directed fisheries and increased shrimp fishing effort in the index zone. Between 2010 and 2012, total allowable catches were increased from 7 million pounds to 8.1 million pounds. Catch quotas were further increased over the years to reflect stock growth, and the 2019 quota was set at 15.1 million pounds (a 65% increase in the quota set in 2007) (SEDAR (Southeast Data Assessment and Review), 2018). In addition, in 2019, management of the recreational Red Snapper fishery was significantly modified through Amendment 50 to expand fishing opportunity for private anglers by delegating some authority from federal managers to individual states (GMFMC (Gulf of Mexico Fishery Management Council), 2019).

In 2011, the level of shrimp effort reduction required in the index area (Figure 2) was decreased from 74% to 67%. This action increased the cap in shrimp trawling in this area to 27,328 days fished, an increase of 5,787 days fished (a 27% increase in allowable effort) (GMFMC (Gulf of Mexico Fishery Management Council), 2007). No further changes in the level of required shrimp trawl effort reductions were made until 2019 when Amendment 18 proposed that effort could be increased by an additional 5,797 days (a 53% increase from the 2007 levels, but still 60% lower than the early 2000s) (GMFMC (Gulf of Mexico Fishery Management Council), 2019). To put this in context of the present US shrimp fishery, there are approximately 1,405 permitted vessels in the fleet (National Marine Fisheries Service, Freedom of Information Act, Selected Gulf of Mexico Shrimp Permit (SPGM) holder records), which would amount to a little over four additional fishing days per vessel.

Prior to such regulatory changes, robust scientific analyses were conducted to quantify the risks to the Red Snapper population at different levels of extraction. In the case of the increased shrimping effort, National Marine Fisheries Service (NMFS) scientists concluded that the proposed increase in bycatch of Red Snapper would reduce the annual allowable catch by no more than 200,000 pounds (∼1% of the present allowable catch) and would not impact the planned schedule to rebuild the Red Snapper stock by 2032 (Goethel and Smith, 2018).

## Pushback against Shrimping

Nonetheless, the narrative that shrimp trawl bycatch is the major threat to Red Snapper is perpetuated in the recreational angling sector, which continues to advocate that regulations should not be relaxed for the shrimp fishery (Venker, 2019, McKinney, 2019). Amendment 18 (recently approved) to the Gulf of Mexico Fishery Management Council's (GMFMC) Shrimp Fishery Management Plan (FMP) allowing increased effort in the shrimp fishery (GMFMC (Gulf of Mexico Fishery Management Council), 2019) generated intense criticisms. A series of articles strongly opposing the proposed rule by mischaracterizing the magnitude of shrimp trawl bycatch in the Gulf of Mexico was published in media outlets targeting recreational anglers (e.g., Sport Fishing Magazine (Venker, 2019, McKinney, 2019) and Texas Saltwater Fishing Magazine (McKinney, 2019)). Erroneous claims included the contention that shrimp trawls catch 7 pounds of fish for every 1 pound of shrimp (McKinney, 2019) (since the mid-2000s bycatch ratios have been closer to 2:1 (Scott-Denton et al., 2012)) and that increasing shrimping effort was projected to “result in the loss of 3.1 million pounds of Red Snapper every year” that could otherwise be harvested in directed fisheries (McKinney, 2019) (rather than no more than 200,000 pounds, annually (Goethel and Smith, 2018)). More broadly, the articles argued that the shrimp fishery was unaccountable, with too little observer coverage to accurately assess their catch of Red Snapper, and that shrimp trawling is inherently destructive and “devastating” to the ocean ecosystem (McKinney, 2019, McKinney, 2019; Venker, 2019).

These articles appeared to be taken at face value by the recreational fishing sector (Olander, 2014) and provided the underpinning for more than 500 formal comments aimed to dissuade NMFS from adopting the rule (public comments available at https://www.regulations.gov/docket?D=NOAA-NMFS-2019-0045). After the comment period ended, a separate article acknowledged the error claiming a loss of 3.1 million pounds of Red Snapper (McKinney, 2019) (although there is no indication of error associated with the original articles), but the overall conclusion that shrimp trawling represents an inherent risk to recreational fishing remained. Despite these efforts to sway NMFS managers, the proposed rule, which was based on rigorous science from the beginning, eventually passed in February of 2020. In that ruling NMFS consolidated the comments into three categories to which they responded, with two categories related to the concerns raised in these articles: allowing an increase in shrimping effort within the Red Snapper index area could (1) lead to unacceptable levels of bycatch of fish, sea turtles, and other species and (2) delay recovery of Red Snapper and lower recreational catch limits in the future. The NMFS responses countered these concerns by re-summarizing the results of their initial analyses (https://www.regulations.gov/document?D=NOAA-NMFS-2019-0045-0599).

## Our View—an Independent Perspective

The management of marine fisheries requires a complex integration of ecological, economic, social, and institutional information with the values of diverse stakeholders (Stephenson et al., 2017). Groups that interact with fish in different ways (e.g., directed fisheries versus bycatch) or for different reasons (e.g., livelihood versus leisure) often diverge in philosophies and perceptions (Johnson and Griffith, 2010). As such, conflicts in shared fishery resources are common. Identifying shared values among stakeholders is a critical aspect of effective management (Barber and Taylor, 1990). A unifying value is that of “sustainability”—that fishing can be maintained in perpetuity without major negative impacts on the fished population or the ecosystem (Pauly et al., 2002, Hilborn et al., 2003). In general, most stakeholders turn to scientists to quantify what amount of harvest is sustainable and to provide guidance on the risks to the population and its ecosystem at different levels of exploitation. Of course, science is iterative and rarely absolute; new and more complete information can alter conclusions dramatically. New science may often be spurred on by challenges from groups who find the initial results at odds with their preferences, beliefs, or experiences. Hypotheses are challenged, new studies are conducted, and the results contribute to better understanding and management of fisheries. Open debate and criticism are a healthy component of the scientific process.

Although science does not solve all management complications (notably, determining the *allocation* of sustainable harvest to different groups), it does allow those debates to occur with all parties understanding the possible ecological implications (Johnson and Griffith, 2010, Gutiérrez and Morgan, 2017). Problems quickly emerge, however, when misinformation is presented as scientific fact—particularly in the context of a broadsided attack by one stakeholder group against another. Although propaganda may be useful to rally troops in war, it does not aide in participatory fisheries management. For the group being attacked the problems are clear—time and resources must be spent countering the claims, public opinion of the group might decline, and less favorable regulations might be enacted. For the group espousing the propaganda the problems may manifest as retaliation by the attacked group, eroded credibility (as the actual facts come to light), and their valid concerns going unaddressed because others simply focus on the erroneous claims (i.e., “crying wolf” never pays off in the long run). The overall result is that the potential for galvanizing divergent viewpoints among groups increases, and the likelihood of reaching consensus is reduced (Johnson and Griffith, 2010). Management of Red Snapper in the US Gulf of Mexico appears poised to tumble further into such a situation.

The Red Snapper stock, although still rebuilding, has improved dramatically allowing for both increases in directed harvest and less restrictive shrimp fishing effort restrictions. It seems that the three main fisheries that catch Red Snapper are being treated in a fair and equitable manner in that each are being allowed to increase harvest levels as the Red Snapper stock grows. Reducing the shrimp fishing cap in the index area to the final level of 60% of the 2001–2003 average for this zone has always been planned to occur when the Red Snapper stock improved to an appropriate level (GMFMC (Gulf of Mexico Fishery Management Council), 2007).

For those in directed fisheries, it is prudent to question what the impacts of increased shrimping might be on their future ability to catch Red Snapper and the broader Gulf of Mexico ecosystem. However, the narrative by some advocates for the recreational sector that the massive reduction in shrimping effort is singularly responsible for the increase in the Red Snapper stock (and thus any increase in effort should be fiercely opposed) is difficult to reconcile with (1) basic knowledge of Red Snapper ecology, (2) the impact of shrimping on Red Snapper relative to the directed fisheries, and (3) the history of Gulf of Mexico shrimping effort. Indeed, the scientific basis of recreational sector's questions were so badly misinformed that, unfortunately, NMFS was able to claim that they had responded by correcting the recreational sector's errors without the need to conduct further scientific inquiry. Thus, the erroneous comments overshadowed the deeper and more substantive concerns, which went unanswered. For instance, despite 79 formal comments (13%) that relayed concerns about potential ecosystem impacts of allowing an increase in shrimping effort, this issue went unaddressed by NMFS (the “real wolf” was allowed to pass). Below, we discuss our views on why we do not believe the allowed increase in shrimping effort will be problematic for Red Snapper and close with a discussion of the concerns raised by recreational fishery advocates that we agree deserve further consideration.

### Red Snapper Ecology

Red Snapper exhibits a life-history common to many marine animals that includes ontogenetic shifts in habitat, whereby younger fish and older fish are spatially segregated (Gallaway et al., 2009, Putman, 2018). Thus, fisheries that operate in different areas interact with different ages of Red Snapper that have different demographic parameters and potential to contribute to stock recovery. Spawning occurs in waters 100–200 m deep, the eggs drift with surface currents for nearly a month before the young settle to the bottom closer to shore and inhabit low-relief habitats. Over the next two years, these fish are subject to high natural mortality rates (86% in the first year of life, 70% in the second year) and bycatch in shrimp trawls. As the fish grow, they move to higher-relief habitat (including rocky outcrops and artificial reefs) where natural mortality rates are much lower (<17%) and become reproductively mature. Shrimp trawlers avoid these habitats, and the risk of bycatch is almost entirely diminished, but at this point Red Snapper enter the directed fisheries. Red Snapper shift to increasingly higher-relief habitat with age until, beginning around 8 years old, they reach a size that largely precludes predation and may expand their range to open bottom habitats (Gallaway et al., 2009, Karnauskas et al., 2017).

Given that high-relief habitats cover a relatively small portion of the Gulf of Mexico (Karnauskas et al., 2017) and that the presence of larger Red Snapper already occupying the structures can prevent the recruitment of smaller fish (Bailey et al., 2001, Workman et al., 2002), there is good reason to suspect survival from ages 2 to 3 years is density dependent (Gazey et al., 2008, Forrest et al., 2013). What this means is that the production or survival of more age-0 and age-1 Red Snapper may not directly translate to an increase of Red Snapper entering the directed fisheries. Support for density-dependent mortality has been reported in the literature for Red Snapper (Gazey et al., 2008, Forrest et al., 2013), but the current stock assessment model fails to adjust for density dependence among juveniles (Lorenzen and Camp, 2019). The consequences of not adjusting for density dependence when it exists is the overestimation of recovery potential and biological reference points such as maximum sustained yield (Forrest et al., 2018). Conversely, including parameters for density-dependent adjustments when they are not needed causes little bias as their estimates approximate zero. In short, including this aspect of ecology in the stock assessment framework for Red Snapper might indicate that the rebuilding schedule for Red Snapper could stall and that further reducing bycatch in young Red Snapper may have limited value.

### Relative Impacts by Directed Fisheries and Shrimping

Large differences exist in how commercial directed fisheries, recreational directed fisheries, and shrimp fisheries interact with Red Snapper. These differences result from Red Snapper ecology and how different fisheries are managed. For instance, “high-grading” is thought to be common in directed fisheries that are managed by limiting the number of fish that can be retained (essentially smaller fish that have been caught are discarded when larger fish are caught (Batsleer et al., 2015)) but would not occur when fisheries are managed by effort limits (Gillis et al., 1995). Regardless of the reason for differences, the relative impact mortality can be difficult to compare among fisheries. We have published a recent paper that attempts to foster communication among commercial, recreational, and shrimp fisheries by weighting Red Snapper catch based on the age structure of catch and natural mortality rates (Putman and Gallaway, 2020). The method converts the number of fish caught to “common age units” so that, for instance, the number of age-1 fish caught in shrimp trawls can be directly compared with the number of age-5 fish caught by recreational fisheries. Applying this approach to data on total catch (bycatch, landings, and discards) from the most recent Red Snapper stock assessment (2005–2015) indicated that shrimp trawls are responsible for <10% of the total catch, commercial directed fishers are responsible for ∼32%, and recreational fishers are responsible for ∼59%. From a management perspective, this finding suggests that the greatest potential to rebuild Red Snapper stocks lies with the directed fisheries, particularly the recreational sector (Putman and Gallaway, 2020).

Likewise, when one considers how each of these three sectors has abided by the regulations of managers, it also appears that room for the most progress lies with the recreational sector. Shrimp trawl bycatch of Red Snapper is managed primarily by an effort cap in the Red Snapper Index Area (Figure 2), whereas the directed fisheries are managed primarily by a landings quota. Shrimping effort has stayed within the required limit each year since the cap was first imposed in 2007. Similarly, the commercial directed fisheries' landings have not exceeded their quota in any year and, based on total catch from 2007 to 2017, this fishing sector was 0.94 million pounds (∼2%) under their quota of 52.02 million pounds. In contrast, the recreational sector exceeded their quota in 8 of the 11 years (2007–2017) with an overage of 17.09 million pounds (∼34%) more than their quota of 50.9 million pounds (GMFMC (Gulf of Mexico Fishery Management Council), 2018). When catch data in the recreational sector were separated between for-hire (e.g., charter boats) and private anglers in 2015, the overage in the recreational sector was entirely attributable to private anglers, as the for-hire sector of the fishery has not exceeded their quota share in any year (GMFMC (Gulf of Mexico Fishery Management Council), 2019). We certainly understand that there are special challenges in effectively managing a diffuse group, and we acknowledge that new efforts are underway (such as individual states managing private anglers through Amendment 50), but we maintain that progress is needed for the private recreational angler component to meet and not exceed their quota share.

### Ecosystem Impacts and the History of Shrimping Effort

To understand the potential ecosystem impacts of allowing shrimping effort to increase, it is important to consider that shrimp fishing effort today in the Gulf of Mexico (even with the proposed increase in the index area) is still considerably lower than it was historically. From 1960 to 1978, shrimp fishing effort in the Gulf of Mexico generally increased from a low of about 100,000 days fished to a high of about 200,000 days fished (Figure 1). Presently, effort ranges from about 63,000 to 75,000 days fished.

The Magnuson-Stevens Act prohibited foreign vessels from fishing in US waters, and other countries responded by prohibiting US vessels from fishing in their waters. This caused US shrimp fishing effort to increase due to the return of US vessels. From about 1978 to 2002, shrimp fishing effort in US waters was, on average, about 200,000 days fished per year (Figure 2). Subsequently, adverse economics (e.g., higher fuel prices and competition from imports) and regulations after 2002 have reduced the present-day, total shrimping effort levels dramatically (∼60%–70%). From 2008 to 2016, Gulf-wide offshore effort has stabilized at 60,000 to 75,000 days fished per year (Figure 2). Likewise, the number of shrimp fishing permits has been limited so that the present-day fleet size does not increase, further ensuring that shrimp fishing effort is maintained at about today's low levels into the future (GMFMC (Gulf of Mexico Fishery Management Council), 2016).

Caution is warranted in making a direct comparison between historical and present fishing effort, as technological efficiency tends to increase the extractive power of fisheries, such that although effort might appear stable the ability to harvest fish is increasing (Palomares and Pauly, 2019). In the case of shrimping, gear improvements, such as turtle excluder devices (TEDs) and BRDs, have actually reversed this typical relationship between time and fishing effort, and the ecological impacts of shrimping for one day are probably less now than ever before (e.g., 40, Raborn et al., 2012). Finfish still dominate the total catches, but the ratio of pounds of fish taken per one pound of shrimp has improved dramatically. In the 1970s, 10 pounds of fish were taken for every pound of shrimp (10:1) (Alverson et al., 1994). However, this ratio has declined to 2:1 since the mid-2000s (Scott-Denton et al., 2012). Why has there been such a decrease in bycatch? NMFS “SEAMAP” (comprehensive annual population surveys) trawl surveys use standard shrimp nets without BRDs and TEDs to provide fishery independent data that can be used to index the population abundance trajectories for shrimp trawl bycatch species. In these surveys, the finfish to shrimp ratio is 16:1 (Southeast Area Monitoring and Assessment Program (SEAMAP)). Thus, the present 2:1 bycatch ratio in commercial shrimping is due mainly to the implementation of BRDs and TEDs.

The pattern of shrimp fishing effort from 1960 to the present implies that whatever impact shrimping is presently having on the Gulf of Mexico ecosystem, it is almost certainly less than it was in the previous decades. Nevertheless, we agree with calls to quantify what those impacts are. One approach that might be taken is to relate spatiotemporal variation in shrimping effort or bycatch to fisheries-independent indices of abundance for ecologically important species. For instance, using SEAMAP data (Southeast Area Monitoring and Assessment Program (SEAMAP)) as long-term indices of juvenile abundance (i.e., recruitment potential) we can examine how certain species have responded to the massive changes in shrimping effort (Figure 1). Specifically, if species are strongly impacted by shrimping one might expect to see an increase in the SEAMAP population index following the sharp decline in effort. Alternatively, if shrimping has no strong impacts on the population dynamics of a species, a relationship between the SEAMAP indices would not be detected.

**Figure 3:**
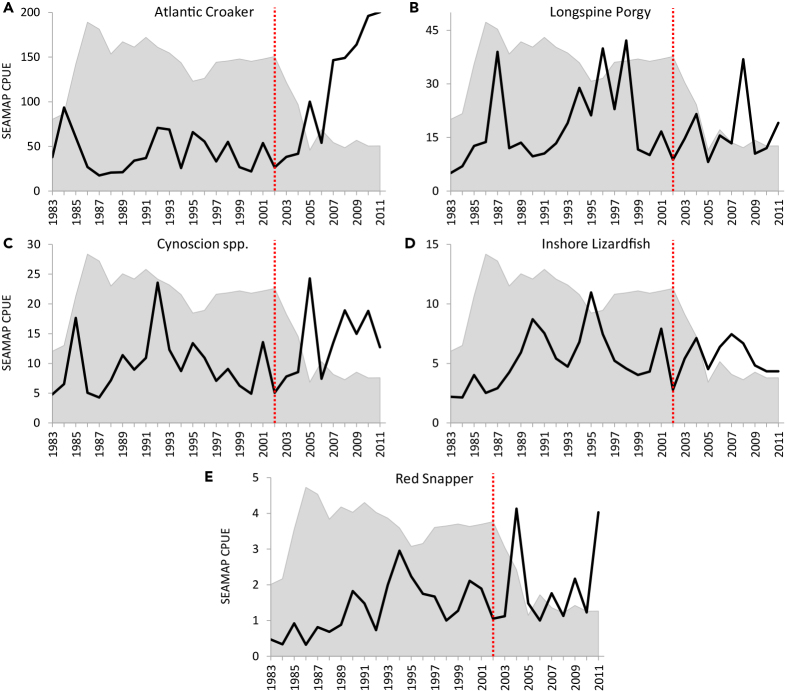
Indices of Fish Abundance and Shrimping Effort Time series of catch-per-unit-effort (CPUE) from the SEAMAP (fisheries-independent) trawl studies (black lines) and relative shrimping effort (shaded area plots; scale not shown). The red vertical line shows the last year of high effort in the Gulf of Mexico shrimp fishery (2002). (A) Atlantic Croaker appears to increase in response to low effort. A somewhat similar but less obvious trend occurs for (C) Cynoscion spp., although with much higher annual variability. No trend and substantial variability throughout the time series is evident for (B) Longspine Porgy and (D) Inshore Lizardfish. There may be a slight increasing trend for (E) Red Snapper over the entire time series irrespective of shrimping effort, but also with high variability.

We compared shrimping effort with indices of abundance for all finfish species caught that constituted ≥5% of the total weight caught in shrimp trawls during the period 1992–2005 (Scott-Denton, 2007) or 2007–2011 (Scott-Denton et al., 2012). These species included Atlantic Croaker (*Micropogonias undulatus*) (representing up to 15.9% of the catch), Longspine porgy (*Stenotomus caprinus*) (representing up to 9% of the catch), Seatrout (*Cynoscion* spp.) (representing up to 5.8% of the catch), and Inshore Lizardfish (*Synodus foetens*) (representing up to 6% of the catch). For comparative purposes we also included Red Snapper, although it only represented 0.3% of the catch. The only clear response was for Atlantic Croaker, which dramatically increased following the drop in shrimping effort (Figure 3).

**Figure 4:**
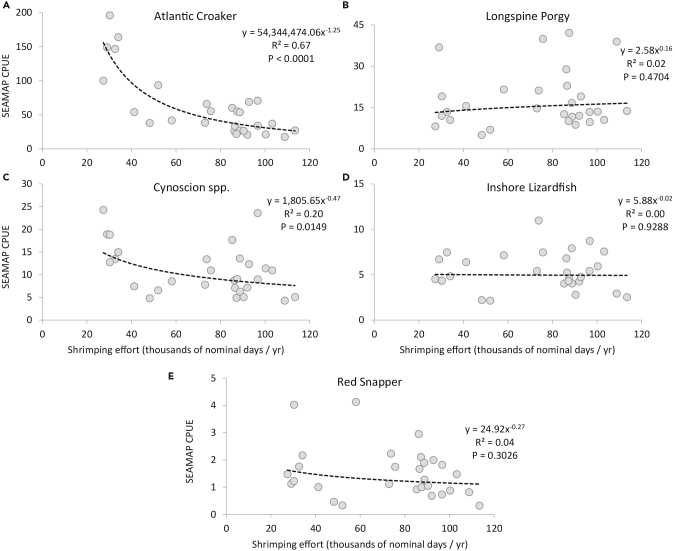
Relationships between Indices of Fish Abundance and Shrimping Effort Catch-per-unit-effort (CPUE) from the SEAMAP (fisheries-independent) trawl studies as a function of shrimping effort. Observed values are given by gray circles; power curve regressions are depicted as black dashed lines. (A) Atlantic Croaker is negatively correlated with shrimping effort (p<0.0001) yielding a relatively high R^2^ = 0.67. (B) Longspine Porgy abundance is unrelated to shrimping effort (p=0.4707). (C) Cynoscion spp. is negatively correlated (p = 0.0149), but with only a moderate amount of variance accounted for, R^2^ = 0.20. (D) Inshore Lizardfish abundance is unrelated to shrimping effort (p = 0.9288), (E) Red Snapper abundance is unrelated to shrimping effort (p=0.3026).

Directly comparing shrimping effort in the areas where SEAMAP indices of abundance were obtained shows a strong negative relationship between Atlantic Croaker abundance and shrimping effort, with shrimping effort accounting for up to 67% of the variation in abundance (Figure 4A). For Seatrout, the relationship was less robust but still detectable, with shrimping effort accounting for 20% of the variation in abundance (Figure 4C). For the other species we examined, no relationship between abundance and shrimping effort was detected. If shrimp trawling constituted a major negative ecosystem-level impact to finfish, one might expect to see negative relationships between indices of fish abundance and shrimping effort even in species that do not constitute a large portion of shrimp trawl catch, such as Red Snapper (Figure 4). Our present analyses suggest that shrimp trawling may have a large, direct impact on some species taken as bycatch (e.g., Atlantic Croaker), but the biomass extracted by shrimp trawls, even at much higher levels than present, does not appear to propagate as indirect effects to finfish species (Figures 3 and 4). Of course, we do not present these findings to take the place of more robust analyses (for instance, application of ecosystem models (deMutsert et al., 2016, Grüss et al., 2016, Walters et al., 2008) but simply use them to illustrate what might be inferred about the impacts of shrimp trawling on the ecosystem.

### Conclusions

We believe that the management of Red Snapper would benefit from less hyperbolic rhetoric. Differences in viewpoints and concerns of risk can be presented in a factual and grounded manner that foster communication among different sectors. We encourage efforts to rebuild the stock to focus on areas where impacts are presently highest, and gains can be most meaningful (i.e., private anglers in the recreational fishery) (Putman and Gallaway, 2020). The shrimp fishery has made many beneficial changes over the years and these changes have been costly to the industry through additional gear requirements and shrimp loss associated with BRDs. We agree that overall bycatch in shrimp trawls should continue to be carefully monitored. The ongoing effort by NOAA's Harvesting Systems Unit and Gear Monitoring Team, Gulf Sea Grant programs, and independent research working collaboratively with the shrimp industry are essential to maintaining gear efficiency and to further quantify and reduce ecosystem level impacts due to shrimping.

## Resource Availability

### Lead Contact

Further information and requests for resources and data should be directed to and will be fulfilled by the Lead Contact, Nathan F. Putman (nathan.putman@gmail.com).

### Materials Availability

This study did not use or generate any reagents.

### Data and Code Availability

The datasets used to support the views presented in this perspectives piece are provided in the Supplemental Information.
