# Supplementary material for: Changes in Shrimping Effort in the Gulf of Mexico and the Impacts to Red Snapper
Source: iScience. 2020 Apr 27;23(5):101111. doi: 10.1016/j.isci.2020.101111 (PMC7240115; doi:10.1016/j.isci.2020.101111)
Supplement: Document S1. Tables S1 and S2 [file mmc1.pdf]

iScience, Volume *23*

Supplemental Information

Changes in Shrimping Effort
in the Gulf of Mexico
and the Impacts to Red Snapper

Benny J. Gallaway, Scott W. Raborn, Laura Picariello, and Nathan F. Putman

Supplemental Information:

Changes in shrimping effort in the Gulf of Mexico and the impacts to Red Snapper

Benny J. Gallaway[1]*, Scott Raborn[1], Laura Picariello[2], & Nathan F. Putman[1]*

1. LGL Ecological Research Associates Inc., Bryan, Texas 77802 USA

2. Texas Sea Grant, Texas A&M University, Corpus Christi, Texas 78412 USA

*Corresponding authors: bjg@lgltex.com (BJG), nathan.putman@gmail.com (NFP)

Datasets used to support the views presented in this perspectives piece are provided in the following tables.

Table S1. Data Associated with Figure 1. Total offshore shrimping effort in the Gulf of Mexico by the U.S. fleet (1960-2016). Annual data are shown as “days fished”, the total number of 24-hour periods of trawling (i.e., each day represents 24 hours of fishing).

Year	Offshore Gulf of Mexico Shrimping Effort (fishing days)
1960	120271.0
1961	85881.0
1962	104318.0
1963	118940.0
1964	128659.0
1965	113778.0
1966	107812.0
1967	126084.0
1968	129541.0
1969	151224.0
1970	127855.0
1971	132102.0
1972	157194.0
1973	146089.0
1974	146415.0
1975	128520.0
1976	154475.0
1977	166307.0
1978	202002.0
1979	211497.0
1980	144256.0
1981	176726.8
1982	173893.8
1983	171310.7
1984	191739.0
1985	196628.2
1986	226797.7
1987	241902.4
1988	205811.6
1989	221164.8
1990	211859.8
1991	223388.4
1992	216668.9
1993	204482.0
1994	195742.1

Year	Offshore Gulf of Mexico Shrimping Effort (fishing days)
1995	176588.5
1996	189653.0
1997	207912.1
1998	216998.6
1999	200474.7
2000	192072.9
2001	197644.4
2002	206620.6
2003	168135.5
2004	146623.9
2005	102839.8
2006	92372.5
2007	80732.8
2008	62797.4
2009	76508.1
2010	60518.3
2011	66777.4
2012	70505.0
2013	64561.4
2014	73682.6
2015	66849.4
2016	72609.1

Table S2. Data associated with Figures 3 and 4. Annual offshore shrimping effort (number of days fished) within the areas sampled by SEAMAP and the corresponding catch per unit effort data (number of fish caught per hour per net) of the fisheries independent survey for Atlantic Croaker (*Micropogonias undulatus*), Longspine porgy (*Stenotomus caprinus*), Inshore Lizardfish (*Synodus foetens*), Seatrout (*Cynoscion spp*.), and Red Snapper (*Lutjanus campechanus*). Raw data available from: https://www.gsmfc.org/seamap-sis.php

Year	Shrimping Effort (days)	Atlantic Croaker	Longspine Porgy	Inshore Lizardfish	Seatrout	Red Snapper
1983	48188.2	38.0	5.1	2.2	4.8	0.5
1984	51996.8	93.5	6.9	2.1	6.5	0.3
1985	85214.2	60.2	12.6	4.0	17.6	0.9
1986	113398.9	26.9	13.7	2.5	5.1	0.3
1987	108776.0	17.5	39.0	2.9	4.3	0.8
1988	92005.6	20.7	12.0	4.3	7.2	0.7
1989	100248.0	21.0	13.5	5.9	11.4	0.9
1990	96692.0	34.0	9.7	8.7	9.0	1.8
1991	103197.3	36.9	10.5	7.5	10.9	1.5
1992	96608.2	70.7	13.4	5.4	23.6	0.7
1993	92731.5	68.9	19.0	4.7	12.3	2.0
1994	86209.3	25.8	28.9	6.8	8.7	3.0
1995	73763.9	66.0	21.2	11.0	13.4	2.2
1996	75657.7	55.4	39.9	7.5	10.9	1.7
1997	86512.2	33.1	22.9	5.2	7.1	1.7
1998	87439.8	55.0	42.1	4.6	9.1	1.0
1999	88749.6	26.8	11.7	4.0	6.3	1.3
2000	87149.4	21.9	10.1	4.3	4.9	2.1
2001	88638.4	53.7	16.7	7.9	13.6	1.9
2002	90338.9	26.5	8.8	2.8	5.1	1.1
2003	72995.9	38.4	14.7	5.4	7.8	1.1
2004	58075.3	41.8	21.6	7.1	8.5	4.1
2005	27392.5	100.1	8.2	4.5	24.3	1.5
2006	41204.3	54.0	15.6	6.4	7.4	1.0
2007	32576.5	146.6	13.3	7.5	13.5	1.8
2008	29067.3	149.0	36.9	6.7	18.9	1.1
2009	34068.3	164.1	10.5	4.8	15.0	2.2
2010	30259.1	196.0	12.0	4.3	18.8	1.2
2011	30326.7	200.4	19.1	4.3	12.7	4.0
